# Evaluation of cardiovascular diseases risk calculators for CVDs prevention and management: scoping review

**DOI:** 10.1186/s12889-022-13944-w

**Published:** 2022-09-14

**Authors:** Mohammed Abd ElFattah Mohammed Darwesh Badawy, Lin Naing, Sofian Johar, Sokking Ong, Hanif Abdul Rahman, Dayangku Siti Nur Ashikin Pengiran Tengah, Chean Lin Chong, Nik Ani Afiqah Tuah

**Affiliations:** 1 PAPRSB Institute of Health Sciences, Universiti Brunei Darussalam, Bandar Seri Begawan, Brunei Darussalam; 2 NCD Prevention Unit, Ministry of Health, Bandar Seri Begawan, Brunei Darussalam; 3 University of Michigan, Ann Arbor, MI USA; 4 Brunei Neuroscience Stroke and Rehabilitation Centre, Pantai Jerudong Specialist Centre, Bandar Seri Begawan, Brunei Darussalam; 5 Raja Isteri Pengiran Anak Saleha Hospital, Cardiology, Bandar Seri Begawan, Brunei Darussalam; 6 Department of Primary Care and Public Health, Imperial College London, London, UK

**Keywords:** Cardiovascular diseases, Risk calculator, Risk assessment, CVD risk, Digital health, Population health

## Abstract

**Background:**

Cardiovascular diseases (CVDs) are the leading cause of morbidity and mortality globally. This review aimed to summarise evidence on the key features, usability and benefits of CVD risk calculators using digital platforms for CVDs prevention and management in populations.

**Methods:**

We used search engines and thematic analyses to conduct a scoping review. As the reporting guideline for this review, we used Preferred Reporting Items for Systematic Reviews and Meta-Analyses extension for Scoping Reviews (PRISMA-ScR).

**Results:**

A total of 17 studies meeting eligibility criteria were included in the analysis, from which about 70% of the studies have prognostic level I (*n* = 8) and level II (*n* = 4) evidence. The review found that various guidelines are recommending different algorithms for CVD risk prediction. The QRISK® was the most accurate CVD risk calculator for several study populations, whereas World Health Organization/International Society of Hypertension (WHO/ISH) risk scores were the least accurate. The key features of CVD risk calculators are variables, predictive accuracy, discrimination index, applicability, understandability, and cost-effectiveness.

**Conclusion:**

For the selected risk prediction tool, development and validation research must be done, which considers a mix of stroke-specific risk and CVD risk to establish its usability in the local community and advantages to the particular health-care environment. To get healthcare professionals more involved in preventing and treating CVDs, each healthcare setting should use an online CVD risk assessment tool that is more useful, accurate, and easy to use, based on the population and health system.

## Background

Cardiovascular disease (CVD) is the primary cause of illness and deaths globally that contribute to enormous healthcare costs. The prevalence of CVD-related deaths is increased from 12.1 million in 1990 to 18.6 million in 2019 and is estimated to reach 24 million by 2030 [1], which results in considerable financial burdens due to high CVD managing costs and the related loss of income. In 2035, CVD will affect more than 130 million people with a total cost of $1.1 trillion [2].

CVD refers to any disorder that can affect the heart and blood vessels, including coronary heart disease, cerebrovascular disease, peripheral arterial disease, rheumatic heart disease, congenital heart disease and deep vein thrombosis [3]. Public health strategies to reduce CVD morbidity and mortality consist of population-level risk factor reduction, individual-based primary prevention and secondary prevention and treatment. Population-level strategies focus on decreasing the whole population’s exposure to CVD risk factors across the life course regardless of the CVD risk, focusing on lifestyle factors. CVD risk refers to the risk of suffering fatal or nonfatal CVD events, for example, the risk of myocardial infarction or stroke in the next ten years [4]. Individual-based primary prevention is targeted at high-risk groups to prevent the onset of CVD through risk factor reduction. The secondary prevention and treatment aim at early detection and treatment to prevent disease progression in people with established CVD [5]. CVD treatments resulted in minimal reductions in risk factors [6]. The ‘vertical’ and ‘total’ cardiovascular risk approaches can mitigate individual CVD risks. The ‘vertical’ approach refers to managing a single risk according to predefined thresholds for treatment initiation, with the presence or absence of levels of concomitant risk factors. The World Health Organization (WHO) recommends the 'total' cardiovascular risk approach in preventing CVD with consideration of healthcare resources, cost-effectiveness and high-risk groups [4]. The approach deems the individual's probability of having fatal or nonfatal cardiovascular events in a given estimated period considering the presence of several predicting risk factors rather than a single risk factor [7].

Several risk calculators directly estimate the outcome of Stroke specifically or as a combined outcome of CVD risk, such as Stroke Riskometer™, a unique tool for assessing the specific risk of Stroke and endorsed by the World Stroke Organization [8]. While other risk calculators are developed to assess the individuals' total CVD risk, the first risk scores were from the Framingham Heart Study (FHS) done in 1976 [9]. The study was based on the western population that may not apply to other populations [10] in developing countries including, Brunei Darussalam.

CVDs such as Ischemic Heart Disease (IHD) and stroke are the top causes of death and significant public health problems in Brunei Darussalam [11]. In 2019, CVDs accounted for 25.5% of all causes of death at an age-standardised rate of 165.5 deaths per 100,000 population [12]. Brunei Darussalam has adapted the CVD risk scoring system from the WHO/ International Society of Hypertension (ISH) chart for the Western Pacific Region A (WPRA) with the absence of evaluation for validity and accuracy in the local population. The Ministry of Health (MOH) Brunei Darussalam introduced the BruHealth mobile application during the COVID-19 pandemic using a digital platform with several key features [13] that have vast impacts on the population health.

The Framingham Risk Score (FRS) is the first CVD risk assessment tool developed about 60 years ago with the concept of primary prevention and estimates a 10-year risk for CVD [14]. The European Society of Cardiology Guidelines in 2016 recommended using the Systematic Coronary Risk Evaluation (SCORE) algorithm [15], based on 12 European cohort studies. It is composed of two distinctive charts for implementation in high and low-risk countries [15]. The American College of Cardiology/American Heart Association (ACC/AHA) Guidelines endorses (ACC/AHA) CVD risk calculator among individuals from 20 to 79 years to detect the high-risk group and predict atherosclerotic cardiovascular disease (ASCVD) as acute myocardial infarction (MI) [16]. The National Institute for Health and Care Excellence (NICE), United Kingdom (UK), advocates the QRISK® risk score and updates it every year [17]. The QRISK® is validated by comparing it against the FRS, and the Scottish ASSIGN scores [18]. The WHO and ISH have jointly developed the WHO/ISH risk prediction charts using data collected from the different regions of WHO sub-regions [19]. The WHO/ISH risk prediction consists of two sets of charts used in settings where blood cholesterol can be measured and settings in which blood cholesterol cannot be measured. The charts categorise individuals into different risk levels [4]. The Stroke Riskometer™ has the aptitude to improve Stroke and NCD prevention markedly. The algorithm is derived from the Framingham Stroke Risk Score (FSRS) prediction based on the INTERSTROKE study. It has performed comparatively poor in predicting stroke events with FSRS and QStroke [20].

There are online cardiovascular risk calculators to measure the probability of developing CVD without defining the appropriate population. Studies evaluate the CVD risk calculators that are clinically effective and cost-effective [21]. This review aims to summarise evidence on CVD risk calculators' key features, usability, and benefits using digital platforms for CVDs prevention and management. Also, we discuss the development and validation process, variables, predictive accuracy, discrimination index, applicability, understandability and cost-effectiveness for CVD risk assessment, in developed and developing countries including Brunei Darussalam.

## Methods of scoping review

**Fig. 1:**
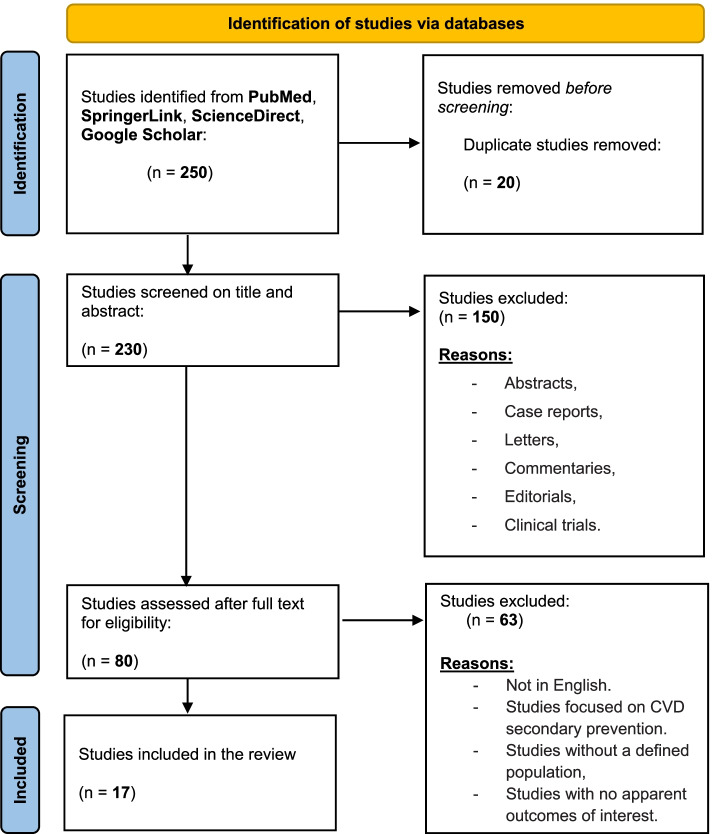
Identification of studies via databases

We conducted a scoping review using the following search engines: PubMed, SpringerLink, ScienceDirect, and Google Scholar. The duration of the search was from 1998 to 2020. The search keywords were combined using Primary Medical Subject Headings (MeSH) and Boolean terms. The main keywords used include “cardiovascular risk assessment”, “CVD risk assessment”, “cardiovascular risk score”, “cardiovascular disease risk score”, “CVD risk score”, “cardiovascular risk calculator”, “cardiovascular disease risk calculator”, “CVD risk calculator”, “cardiovascular risk”, “coronary risk score”, “risk equation”, “risk scoring method”, “risk prediction”, “risk algorithms”, “QRISK” and “WHO/ISH”. The type of included articles are reviews and observational studies. We used Preferred Reporting Items for Systematic reviews and Meta-Analyses extension for Scoping Reviews (PRISMA-ScR) in the review (Fig. 1) [22]. The initial literature search of this review yielded about 230 eligible abstracts. We analysed the abstracts of the publications and excluded abstracts, case reports, letters, commentaries and clinical trials. We reviewed 80 full-text articles and only included 17 articles in the final analysis that met the eligibility inclusion criteria of this review, which primarily included studies published only in the English, focused on CVD risk calculators for primary CVDs prevention and management, and provided evidence on CVD risk calculators with defined population and apparent outcomes of interest.

## Results

Table 1 shows the characteristics of included studies on CVD risk calculators. The included studies can be categorised into Level I (*n* = 8), II (*n* = 4) and IV (*n* = 5) based on the levels of evidence for prognostic studies [23].

**Table 1:** Characteristics of included studies on CVD risk calculators

Authors/ year	CVD calculators	Study design	Level of evidence
Mendis et al. / 2007 [19]	WHO/ISH risk charts	A Hypothetical cohort for different 14 sub-regions	Level II prognostic study
Hippisley-Cox et al. / 2007 [10]	Framingham risk score QRISK1**®** ASSIGN	A Prospective open cohort study	Level I prognostic study
Beswick et al. / 2008 [24]	A total of 70 risk scoring methods	Series of systematic reviews	Level I prognostic study
Hippisley-Cox et al. / 2008 [18]	Framingham risk score QRISK1**®** QRISK2**®**	A Prospective open cohort study	Level I prognostic study
Collins et al. / 2009 [25]	Framingham risk score QRISK1**®**	A Prospective open cohort study	Level I prognostic study
Collins et al. / 2010 [17]	Framingham risk score QRISK1**® &** QRISK2**®**	A Prospective open cohort study	Level I prognostic study
Mendis et al. / 2011 [26]	WHO/ISH risk charts	A Cross-sectional population-based survey	Level IV prognostic study
Siontis et al. / 2012 [27]	Framingham risk score ASSIGN SCORE PROCAM Reynolds risk score QRISK1® & QRISK2®	A Systematic review of comparative predictive model studies	Level I prognostic study
Kariuki et al. / 2013 [28]	Framingham non-laboratory Gaziano non-laboratory WHO/ISH non-laboratory Swedish Consultation-based UK General Practice model	A Systematic review of non-laboratory-based CVD algorithms	Level I prognostic study
Otgontuya et al. / 2013 [29]	WHO/ISH risk charts	A Retrospective cohort study in 3 Asian countries	Level II prognostic study
Bansal et al. / 2014 [30]	JBS3 CVD risk calculator ACC/AHA ASCVD) Risk Framingham risk score WHO/ISH risk charts	A Cross-sectional study	Level IV prognostic study
Selvarajah et al. / 2014 [31]	Framingham risk score SCORE-high and -low WHO/ISH risk charts	A Retrospective cohort study	Level II prognostic study
Ofori et al. / 2017 [32]	ACC/AHA ASCVD) Risk Framingham risk score WHO/ISH risk charts	A Cross-sectional study	Level IV prognostic study
Bonner et al. / 2018 [33]	73 unique CVD risk calculators	A Systematic review to measure the validity, understandability, and actionability of online CVD risk calculators	Level I prognostic study
Kaptoge et al. / 2019 [34]	2019 WHO risk charts	A Retrospective cohort study from 21 global regions	Level II prognostic study
Hasabullah et al. / 2020 [21]	Framingham risk score SCORE ACC/AHA ASCVD) Risk QRISK®	A Cross-sectional study	Level IV prognostic study
Hosein et al. / 2020 [35]	Framingham risk score ASSIGN QRISK2®	A Cross-sectional study	Level IV prognostic study

### Summary of existing CVD risk calculators

The CVD risk calculators widely investigated in studies include FRS, SCORE, ACC/AHA (ASCVD) Risk Estimator, QRISK® and WHO/ISH risk prediction charts [36]. The typical evaluation method for CVD risk calculators comprises a sample of local patients without prior CVD and with a presence of acute MI. Then, individuals apply the different calculators to estimate the predicted 10-year risk of CVD events if presented just before suffering the acute MI [21]. QRISK® is the most accurate CVD risk calculator for several study populations. A study conducted in Saudi Arabia states high-risk estimates for each calculator, including ACC/AHA (44.2%), Euro-SCORE (22.5%), FRS (29.5%), and QRISK® (95.3%). The QRISK® is the most accurate CVD risk assessment tool besides being most applicable for the Saudi population [21]. Another study in India showed higher risks for the Joint British Societies (JBS), FRS and ACC/AHA instruments (55.9%, 38.3% and 30.2%, respectively) than the WHO/ISH risk scores (13.4%) [30]. The ACC/AHA scored 50.2% as the most helpful guide for initiating statin therapy for primary prevention of CVD and 16.9% for the FRS, and 15.2% for the WHO/ISH risk chart in the Indian population. The WHO/ISH risk scores are the least accurate CVD risk assessment tool [32].

Table 2 compares QRISK® and WHO/ISH risk calculators. The WHO utilizes a hypothetical dataset for each of the six regions based on the prevalence of risk factors discovered by a previous Collaborative Risk Assessment Project [37] The WHO/ISH model regression equations have not been released for academic or clinical use [19]. The QRISK® was created using Cox proportional hazards from a large UK cohort database [18]. The QRISK® CVD risk calculators were checked internally and externally by a number of studies with different ratings for ethnicity and poverty [38].

**Table 2:** Comparison of characteristics between QRISK® risk calculator and WHO/ISH risk charts

Characteristics	QRISK®	WHO/ISH risk charts
Derivation study	Prospective cohort database (version 19 of the QRESEARCH database)	Hypothetical cohorts for different 14 sub-regions derived from the WHO Comparative Risk Assessment study [37]
Statistical method	Cox proportional hazards models	Non- specified Modelling approach
Population of study	2.29 million patients	Not applicable
Sample size	1,535, 583 patients (Simple random sampling)	Not applicable
Duration of the study	1 January 1993 to 31 March 2008	2007
Age of participants	**35—75**	40—79
Country of validation	United Kingdom	Not applicable
Ethnicity involved	White/not-recorded, Indian, Pakistani, Bangladeshi, other-Asian, black-African, black-Caribbean, Chinese, Mixed	Not involved
Number of variables	**14**	**6**
Type of variable	• Fixed Risk Factors • Modifiable Risk Factors • Ongoing Clinical Conditions	• Fixed Risk Factors (Age, sex) • Modifiable Risk Factors + Diabetes
Applicability and ease of use	• Online available calculator • Clinical and lab variables are needed for more accurate personalized risk	Two paper-based versions of WHO/ISH risk charts for each sub-region
Discrimination in AUROC	**0.792**	**0.613** (Discrimination in Asian population)
10-year risk estimate	Percentage & Categories	Categories
Risk categories	• < **5%,** • **5- 10%,** • **10–20%,** • > **20%**	• **Green** < 10% • **Yellow** 10% to < 20% • **Orange** 20% to < 30% • **Red** 30% to < 40% • **Deep Red** > 40%
Costs involved	• Involve more costs for lab and clinical examination required for complete risk assessment	• Fewer costs are involved, especially for charts without blood cholesterol investigation

Table 3 compares QRISK® and WHO/ISH risk calculators. The QRISK® risk calculator is a CVD risk score that is dynamically updated from anonymized e-health records to reflect changes in the population. The WHO/ISH risk charts, on the other hand, can be used in healthcare settings with limited resources because they use simple variables. Due to the submission of CVD risk variables, some QRISK® data are missing. An estimated score is made using previously recorded data and expected values based on ethnicity, age, and sex. WHO/ISH risk charts will only apply to the region's most populous country.

**Table 3:** Comparison of advantages and disadvantages between QRISK® risk calculator and WHO/ISH risk charts

	**QRISK®**	**WHO/ISH risk charts**
Advantages	1. Dynamically updated CVD risk score developed from annually updated anonymized e-health records to reflect changes in the population characteristics 2. Various ethnicity and deprivation groups are included in calculating the CVD risk score for population groups most likely disadvantaged by the other risk algorithms 3. Additional modifiable risk factors and ongoing clinical conditions are included to quantify the CVD risk score for every individual patient 4. CVD Risk scores could be saved through digital online platforms if embedded within mobile or computer applications 5. Upgradeable to a national comprehensive CVD risk factors profile and a rank-ordered recall list	1. Charts can be used even in resource-constraint healthcare settings due to simple variables 2. Allow improvement and effectiveness of CVD risk assessment even in the countries that do not possess sophisticated technology for the use of online calculators through established health information systems 3. The WHO/ISH risk charts provide an optimal visual illustration when explaining the implications of elevated CVD risk to the patients through colour-coded CVD risk categories [24]
Disadvantages	1. Some data are usually missing due to the submission of several CVD risk factors; an estimated QRISK® score is usually calculated using the previously recorded data and predicted values based on the patient's ethnicity, age, and sex 2. High costs are needed for incorporating into a primary health setting with an appropriate computer system and costly screening investigations and examinations are needed for complete “actual” QRISK® CVD risk assessment 3. Patients presented with a QRISK® score acknowledged their CVD risk level, but it is usually unclear if they correctly understood the 10-year CVD percentage risk. It needs after-score recommendations and lifestyle modifications to prevent the poor patient recall of CVD risk, confusion or misunderstanding [39]	1. Results will be only applicable to the country with the largest population in the region 2. Charts underestimate the risk in several groups of people, e.g. Hypertensive with blood pressure persistently ≥ 160/100 mm Hg, people with blood cholesterol ≥ 8 mmol/l, diabetics, people with a history of diagnosed ischemic heart disease or renal patients [40] 3. The WHO/ISH risk charts often incorrectly categorize most people into the low CVD risk group due to no prior validation, leading to a higher under-treatment rate and more complications and costs to incur [31] 4. The over-simplification and absence of information on validation or discrimination index have a real effect on the sensitivity, specificity and predictive accuracy of the WHO/ISH risk charts [31]

Table 4 compares the variables of QRISK®2 and WHO/ISH. Gender, age, systolic blood pressure (SBP), diabetes, total cholesterol (mmol/l), and smoking status were variables in the WHO/ISH CVD risk chart [4]. The QRISK® risk score takes into account your ethnicity, family history (angina or heart attack in a first-degree relative younger than 60 years), cholesterol/HDL ratio, BMI, hypertensive medication, rheumatoid arthritis, chronic kidney disease, and atrial fibrillation [17, 18]. Table 5 compares CVD risk assessment tools' discrimination. QRISK® outperformed other CVD risk calculators. Discrimination performance is based on the area under the curve (AUC) metric [10, 17, 18, 25].

**Table 4:** Comparison of variables for QRISK®2 risk calculator and WHO/ISH risk charts

*Variables*		QRISK®2	WHO/ISH
**Fixed Risk Factors**	Age	X	X
	Gender	X	X
	Ethnicity	X	–
	Relevant family history	X	–
**Modifiable Risk Factors**	Smoking status	X	X
	Systolic blood pressure	X	X
	HDL Cholesterol level	X	–
	Total Cholesterol Level	X	X
	Body mass index	X	–
	Deprivation score	X	–
**Ongoing clinical Conditions**	Ongoing Diabetes	X	X
	Ongoing hypertensive medication	X	–
	Ongoing Rheumatoid arthritis	X	–
	Ongoing chronic kidney disease	X	–
	Ongoing Atrial Fibrillation	X	–

**Table 5:** Discrimination performance of CVD risk assessment tools according to (AUC) metric

		**AUC (95% CI)**	
Study	**Models**	**Men**	**Women**
**Collins et al. (2010) **[17]	Framingham risk score	**0.75** (0.747 to 0.753)	**0.774** (0.771 to 0.777)
	QRISK1**®**	**0.771** (0.768 to 0.774)	**0.799** (0.796 to 0.802)
	QRISK2**®**	**0.773** (0.770 to 0.776)	**0.801** (0.798 to 0.804)
**Collins et al. (2009) **[25]	Framingham risk score	**0.752** (0.749 to 0.755)	**0.770** (0.766 to 0.774)
	QRISK1**®**	**0.762** (0.759 to 0.765)	**0.789** (0.785 to 0.793)
**Hippisley-Cox et al. (2008) **[18]	Framingham risk score	**0.779** (0.776 to 0.782)	**0.800** (0.797 to 0.803)
	QRISK1**®**	**0.788** (0.786 to 0.791)	**0.814** (0.811 to 0.817)
	QRISK2**®**	**0.792** (0.789 to 0.794)	**0.817** (0.814 to 0.820)
**Hippisley-Cox et al. (2007) **[10]	Framingham risk score	**0.759** (0.756 to 0.764)	**0.774** (0.771 to 0.778)
	QRISK1**®**	**0.767** (0.763 to 0.772)	**0.788** (0.785 to 0.791)
	ASSIGN	**0.764** (0.760 to 0.769)	**0.784** (0.781 to 0.787)

## Discussion

### Key features of CVD risk calculators

#### Development and validation

The WHO/ISH risk prediction charts seem the only option available for the populations for which prospective studies are not available [30]. The WHO CVD Risk Chart Working Group updated the WHO CVD risk charts in 2019 based on newly validated risk prediction models using Cox proportional hazards models to estimate CVD risk in 21 Global Burden of Disease (GBD) regions [34]. It utilised data from 85 prospective cohorts based on the Emerging Risk Factors Collaboration (ERFC). The charts are recalibrated using data from the GBD studies and the Non-Communicable Disease Risk Factor Collaboration (NCD-RISC) and externally validated using data from a further 19 prospective cohorts [34]. Evidence suggests that the updated WHO CVD Risk Charts are not formally endorsed and not widely applied, except in a study done among a cohort of Bangladeshi adults [41]. The study's findings stated that the charts could enhance the accuracy, practicability, and sustainability of efforts to reduce the burden of CVDs [34]. It is mentioned above that the QRISK® was developed from an extensive UK cohort database and the statistical method used was the Cox proportional hazards. Also, multiple imputation statistical techniques allow patients with incomplete data included in analyses, enable full use of all the available data, and increase power and precision [18]. Also, QRISK® CVD risk calculators were internally and externally validated by several studies with independent contributions of ethnicity and deprivation scores. However, Asians in the QRISK cohort are only 4.1% and 3.6% of the total population in the derivation and validation cohorts. It is updated annually to reflect the population changes [38].

#### Variables

It is highlighted in Table 4 that the WHO/ISH CVD risk chart variables were gender, age, systolic blood pressure (SBP), presence or absence of diabetes, total cholesterol level (mmol/l), and smoking status [4]. The QRISK® risk score incorporates more variables including ethnicity, relevant family history (angina or heart attack in a first-degree relative younger than 60 years old), cholesterol/HDL ratio, body mass index, hypertensive medication, rheumatoid arthritis, chronic kidney disease and atrial fibrillation [17, 18]. Nevertheless, a shortage of cohort studies analysing CVD risk and weighting such variables in different populations, particularly the Asian population, led to the poor discrimination between observed CVD events and estimated CVD risk in Asians [42]. The updated QRISK®3 risk prediction models were developed with the inclusion of additional clinical variables. The variables include chronic kidney diseases (including stage 3 CKD), systolic blood pressure, migraine, corticosteroids and systemic Lupus Erythematosus. It enables doctors to identify those at most risk of heart disease and stroke [43].

#### Predictive accuracy

Studies showed that the WHO/ISH risk prediction model identifies most people with low CVD risk, for instance, 97% (95% CI 96.4, 97.7) for Cambodia, 89.6% (95%CI 86.8, 92.2) for Mongolia and 94.4% (95%CI 91, 97.8) for Malaysia [29]. The prevalence of low CVD risk was 89.3% [44] in Jamaica and 89.7% in Cuba [45]. Another study reported that the prevalence of CVD risk factors was high, but it did not translate into high CVD risk categorisation [31]. In addition, the prevalence of high total CVD risk was estimated to be less than 10% in people aged 40 or over in China (1.1%), Iran (1.7%), Sri Lanka (2.2%), Cuba (2.8%), Nigeria (5.0%,) Georgia (9.6%) and Pakistan (10.0%) [26]. It is plausible that the QRISK® risk calculator demonstrated more prediction accuracy than other CVD risk assessment instruments (as shown in Table 5). The discrimination performance is based on the area under the curve (AUC) statistic and identifies individuals who will experience an event [10, 17, 18, 25]. A recent study among multi-ethnic Caribbean individuals demonstrated that the QRISK®2 risk prediction model (AUROC = 0.96) is superior to ASSIGN (AUROC = 0.93) and the Framingham risk prediction model (AUROC = 0.92) [35].

#### Applicability and understandability

It is stated in Table 2 about the applicability and understandability of the CVD risk calculators, that clinicians often used the WHO/ISH risk charts for quick and consistent estimation of total CVD risk in 'individuals' [46]. The charts provide an optimal visual aid when explaining the implications of elevated risk and treatment options. In the primary care settings, the charts are likely preferred due to their simplicity to patients and physicians and applicability, especially in low-resource settings where online risk calculators could be complex due to technological challenges [24]. The QRISK® risk calculator is an online CVD risk algorithm and is integrated into clinical management systems. It generates an estimated score based on existing data to evolve as data quality and completeness improve and population characteristics change [18]. Some patients might not understand the meaning or the significance of some CVD risk factors. It may make assumptions about missing data, leading to less accurate results [39]. The JBS recommendations on preventing CVD introduced the JBS3 risk calculator in 2014, focusing on lifetime risk. It uses various visual displays and other metrics, for example, "Heart Age". The JBS3 has main advantages over QRISK®, primarily having multiple ways of presenting risk information that may accommodate the needs and preferences of a range of patients and can facilitate practitioner communication [47].

#### Cost-effectiveness

The WHO/ISH risk charts can be used in low-resource healthcare settings as part of stepwise approaches to help target laboratory testing. Also, individuals most likely benefit from the extra information and can use the charts even when values for some risk factors are unavailable [19]. In contrast, the risk charts incorrectly categorised most people into the low CVD risk group due to no prior validation study done, leading to higher rates of under-treatment and subsequently more complications and cost spending [31]. The CVD risk assessment tools can identify patients for CVD prevention in primary care opportunistically or through active CVD risk assessment [15]. A study done in the UK reported CVD preventive measures using the QRISK® algorithm among 40–74 years individuals were highly cost-effective compared with opportunistic assessment [48]. Conversely, the QRISK® risk calculator implemented in the primary health setting will require spending high costs for the appropriate computer system, screening investigations and examinations for a complete QRISK® CVD risk assessment [18].

### CVD risk calculators in Asia and Brunei Darussalam

The existing CVD risk-assessment tools are not universal due to genetic differences, cultures, lifestyle habits, and social and behavioural characteristics [49]. The Asia Pacific Cohort Studies Collaboration showed higher systolic blood pressure, total cholesterol, and CVD events in Framingham than in the Asian cohorts. Smoking is higher in the Asian cohort [50]. The FRS has overestimated the risk in the Asian population [51]. There is limited evidence on the most effective CVD risk calculator for risk stratification in Asian populations, including Brunei Darussalam. It is appropriate to develop a predictive equation using data obtained from a representative and contemporary cohort of a population [52]. Some health systems develop their stroke-specific risk calculator based on their unique population and apply it to primary prevention for populations without a history of cerebrovascular disease. However, some develop calculators to predict Stroke in atrial fibrillation, recent transient ischemic attack or history of previous stroke [8].

The WHO/ISH risk prediction chart for WPRA is the algorithm adopted for CVD risk assessment among individuals in Brunei Darussalam [11]. The charts have not been validated in Brunei Darussalam due to the absence of prospective cohort studies [53]. This risk prediction chart might have underestimated the total CVD risk in the Bruneian population due to antihypertensive therapy, as noted in the WHO/ISH risk charts guidelines [54]. Health care professionals in Brunei Darussalam must consider the key features of the CVD risk calculator and carry out external validation of the tool to assess its feasibility and effectiveness. Besides, they must consider the predictive accuracy of using the calculator to ensure its beneficial outcome tool for the population.

### Recommendations

The countries that need to develop national CVD risk calculators or plan to use one of the currently available CVD risk assessment tools should consider the key features that could affect the validity and accuracy of the calculator in determining the usability and benefits of the tool in its respective health care settings. Also, there is a need to consider the development and validation study of the tool, which considers a combination of stroke-specific risk with CVD risk. The key features are variables, predictive accuracy, discrimination index, applicability, understandability, and cost-effectiveness. For Brunei Darussalam, the digital deployment of the QRISK®3 or JBS3 CVD risk calculator through the national 'BruHealth' mobile application may be feasible and applicable to assess CVD risks in the population. In addition, the use of digital machine learning and laboratory measurements could provide more reliable predictive accuracy than the WHO/ISH risk charts. Health care professionals should consider the characteristics of a population in determining the most feasible and accurate tools for the respective health system. Research studies should be conducted focusing on the validation and evaluation (usability and feasibility) of a CVD risk calculator for a particular population, utilising comparative evidence for the CVD risk calculators.

## Conclusion

We found that various guidelines are recommending different algorithms for CVD risk prediction. The QRISK® was found to be the more accurate CVD risk calculator for several study populations, whereas WHO/ISH risk scores were discovered to be the least accurate. The key features of CVD risk calculators are variables, predictive accuracy, discrimination index, applicability, understandability, and cost-effectiveness. Also, it is valuable to integrate stoke-specific risk assessment in the CVD risk calculator. A development and validation study must be conducted for the selected risk prediction tool to determine its usability to the local population and benefits to the respective health care setting. Overall, each health care setting should utilize a more feasible, accurate and user-friendly online CVD risk assessment tool tailored to the population and health system. Future research should focus on the validation and evaluation methods of the digital CVD risk calculators to assess the feasibility and benefits of tools to the respective populations.

### Strengths and limitations

Our scoping review has several strengths, including the fact that it is the first scoping review on evidence related to CVD risk calculators' key features, usability, and benefits when used with digital platforms for CVD prevention and management. We also made the scoping review process transparent by using a clear search methodology that referred to the level of evidence for prognostic studies for each study included in the review, as well as explicit inclusion and exclusion criteria. The key limitations are the lack of a critical evaluation of the included studies and little bias assessment, as well as the use of search engines rather than research databases to broaden the search area.

## Data Availability

All data analysed during this study and supporting its findings are included in this published article and all studies included in this review are available in the table (1).

## Abbreviations

ACC/AHA: American College of Cardiology/American Heart Association
ASCVD: Atherosclerotic cardiovascular disease
AUROC: Area Under the Receiver Operating Characteristic curve
CVDs: Cardiovascular diseases
FHS: Framingham Heart Study
FRS: Framingham Risk Score
IHD: Ischemic Heart Disease
ISH: International Society of Hypertension
MOH: Ministry of Health
NCD: Non-communicable diseases
NICE: National Institute for Health and Care Excellence
PRISMA-ScR: Preferred Reporting Items for Systematic Reviews and Meta-Analyses extension for Scoping Reviews
SCORE: Systematic Coronary Risk Evaluation
WHO: World Health Organization
WPR-A: Western Pacific Region A
